# Supplementary material for: ‘Share your views’—international consultation informs a patient engagement strategy for the Multinational Association of Supportive Care in Cancer
Source: Support Care Cancer. 2022 Oct 10;30(12):9953–61. doi: 10.1007/s00520-022-07366-y (PMC9548426; doi:10.1007/s00520-022-07366-y)
Supplement: Supplementary file 1 — Supplementary file1 (DOCX 414 KB) [file 520_2022_7366_MOESM1_ESM.docx]

**Figure S1: Terminology preferences categorized by (A) age and (B) gender.** **(A)** Overall preferred terminologies in absolute numbers (*n*), stratified by age. **(B)** Overall preferred terminologies in absolute numbers (*n*), stratified by gender.

| **Table S1: Terminology preferences ranking and scores** | | | | | | | | | | |
| --- | --- | --- | --- | --- | --- | --- | --- | --- | --- | --- |
| **Preference** | **1** | **2** | **3** | **4** | **5** | **6** | **7** | **8** | **TOTAL** | **SCORE** |
| **Patient** | 58.64%  173 | 16.95%  50 | 5.42%  16 | 5.76%  17 | 2.37%  7 | 3.39%  10 | 3.05%  9 | 4.41%  13 | 295 | 6.79 |
| **Consumer** | 2.31%  6 | 14.23%  37 | 10.00%  26 | 11.54%  30 | 5.77%  15 | 12.31%  32 | 23.46%  61 | 20.38%  53 | 260 | 3.63 |
| **End user** | 0.78%  2 | 2.35%  6 | 9.02%  23 | 7.45%  19 | 10.20%  26 | 9.02%  23 | 23.53%  60 | 37.65%  96 | 255 | 2.67 |
| **Community** | 1.92%  5 | 9.23%  24 | 19.62%  51 | 21.15%  55 | 22.31%  58 | 17.31%  45 | 6.15%  16 | 2.31%  6 | 260 | 4.59 |
| **People affected by cancer** | 26.55%  77 | 35.17%  102 | 14.48%  42 | 6.90%  20 | 7.93%  23 | 3.10%  9 | 2.76%  8 | 3.10%  9 | 290 | 6.30 |
| **Public** | 2.32%  6 | 5.41%  14 | 9.27%  24 | 14.67%  38 | 20.08%  52 | 29.34%  76 | 11.58%  30 | 7.34%  19 | 259 | 3.84 |
| **Advocate** | 7.78%  21 | 14.44%  39 | 20.00%  54 | 14.07%  38 | 15.93%  43 | 10.00%  27 | 12.96%  35 | 4.81%  13 | 270 | 4.78 |
| **Carer** | 6.04%  16 | 6.79%  18 | 15.09%  40 | 15.85%  42 | 11.32%  30 | 13.21%  35 | 13.21%  35 | 18.49%  49 | 265 | 3.95 |
