# Supplementary material for: ‘Share your views’—international consultation informs a patient engagement strategy for the Multinational Association of Supportive Care in Cancer
Source: Support Care Cancer. 2022 Oct 10;30(12):9953–61. doi: 10.1007/s00520-022-07366-y (PMC9548426; doi:10.1007/s00520-022-07366-y)
Supplement: Supplementary file 2 — Supplementary file2 (DOCX 950 KB) [file 520_2022_7366_MOESM2_ESM.docx]

**‘Share your views’ - international consultation informs a patient engagement strategy for the Multinational Association of Supportive Care in Cancer**

Hannah R. Wardill, PhD^1,2^*, Yin Ting Cheung, PhD^3^*, Anna Boltong, PhD^4^, Andreas Charalambous, RN, PhD^5,6^, Bogda Koczwara, BM BS FRACP^7^, Maryam Lustberg, MD, MPH^8,9^, Elaine Tomlins, RN, PhD^10^, Joanne M. Britto, PhD^11^.

1. School of Biomedicine, The University of Adelaide, Adelaide, South Australia, Australia
2. Supportive Oncology Research Group, Precision Medicine Theme (Cancer), South Australian Health and Medical Research Institute, South Australia, Australia
3. School of Pharmacy, Faculty of Medicine, The Chinese University of Hong Kong, Hong Kong SAR
4. Cancer Australia, Sydney, Australia
5. Cyprus University of Technology, Cyprus
6. University of Turku, Finland
7. Flinders Medical Centre and Flinders University, Adelaide, Australia
8. Section of Medical Oncology, Department of Medicine, Yale School of Medicine, New Haven, CT, United States
9. Yale Cancer Center, New Haven, CT, United States
10. Royal Marsden NHS Foundation Trust London UK
11. Victorian Comprehensive Cancer Centre Alliance, Melbourne, Australia

** Denotes equal authorship*

Running Title: MASCC-led international survey on consumer engagement

Corresponding Author:

Dr Joanne M Britto

Manager, Consumer Involvement

Victorian Comprehensive Cancer Centre Alliance

Melbourne, Victoria, Australia

+61 3 8559 9055

joanne.britto@unimelb.edu.au

**Key Words**: patient and public advocacy, consumer and community involvement, people affected by cancer, supportive cancer care, terminology

**Disclosures**

All authors have no conflict of interest to disclose

**Acknowledgements**

The authors would like to acknowledge members of the VCCC Alliance Consumer Network for review of the survey, Susan McCarthy for her assistance on this project, and organizations and individuals who helped distribute the survey for multinational reach.

**Abstract**

**Introduction**. Engaging with patients and public (consumers and community) enhances the relevance of cancer control developments, however, challenges remain to integrate into processes. Medical and other professional societies are well-positioned to foster and endorse best-practice.

**Methods**: Between October-December 2021, the Multinational Association of Supportive Care in Cancer (MASCC) conducted a global consultation with those who identified as “people affected by cancer”. Recruitment to an online cross-sectional survey was by a combination of purposive and convenience sampling to determine preferred terminologies and experiences with MASCC and other cancer-related societies.

**Results**: The survey was completed by 343 respondents from 29 countries, a majority being female (78.2%) and younger than 60 years of age (62.1%). Respondents preferred to be identified as ‘patient’ from a set of defined terms, however, this only accounted for 49% - 67% selected response across geographical regions. Only 22.2% of respondents had engaged previously with MASCC, of whom 90.8% reported a positive experience through involvement with education and information, networking and collaboration, and practice guidelines. Respondents perceived areas of opportunity as: early involvement in decision making, educational initiatives, open communication, and information sharing. Across all geographical regions, responders chose a preference to contribute to future consumer research (53.0%), policy (31.7%) or consumer engagement activities (56.9%) including participation in a conference session (65.0%) or patient day (47.9%).

**Conclusions**: This survey provides a first insight into how consumers wish to engage with MASCC. These values will be embedded into a strategy that aims for effective and sustainable partnerships with multinational consumers.

**Introduction**

Quality engagement with patients and the public (herein termed consumers) benefits the community and those involved in cancer control. Collective evidence has demonstrated that incorporating the lived experience is effective in improving the quality and safety of healthcare [1-3]. Over recent years, the translation of research into diagnostics and treatment [4-6], as well as emphasis on delivering quality supportive care [7-9], suggests momentum change to increase the prominence of the consumer’s perspective in research and policy development for cancer care.

At both an organizational and policy level, there is world-wide recognition for valuing these key stakeholders by governments, research institutes and funding agencies. Consumer involvement is highly recommended in every stage of decision-making processes, such as developing healthcare policies [10], investigating the evidence of impact [11], setting research priorities, and disseminating research findings [12]. However, the continual challenge remains to embed engagement strategies into health service and research practice [13].

Healthcare Societies and Associations play an important part in reinforcing an effective role for consumers. The Multinational Association for Supportive Care in Cancer (MASCC), an international, multidisciplinary organization, is dedicated to research and education in all aspects of supportive cancer care. Appreciating that supportive care needs for individuals are unique and best understood and explained by those who have or are experiencing cancer, it is recognized that consumers are not only research participants but have a potential role in informing research priorities and governance [14]. With the overarching aim of promoting consumer engagement in supportive care, the MASCC Consumer Engagement Committee was formed in February 2021. The role of the Committee extends to progressing strategic directions in increasing awareness of supportive care and facilitating different levels of consumer involvement in MASCC’s strategy and operations.

For MASCC to develop a consumer engagement strategy with international adaptation, it is necessary to use terminology that individuals identify with to improve uptake and effective participation [15, 16]. An absence of consistent nomenclature existing across geographical distinctions is demonstrated with references to a ‘consumer’, ‘patient advocate’ or equivalent term. The term ‘patient and public’ used in the UK, US, and Canada includes patients, service users, carers and families and the general public [10]. In Australia and New Zealand, consumer refers to patients and potential patients, carers, and people who use healthcare services; community is a group of people sharing a common interest (e.g. cultural, social, political, health, economic interests) but not necessarily a particular geographic association [12]. Other countries or societies may use equivalent terms or may not have an existing term to describe patient engagement beyond a recipient of healthcare. Notably, in a systematic review to examine the experiences, outcomes and quality of recent patient and public involvement in cancer research, none of the 27 included studies were conducted in Asia, Africa and Latin America [4]. Given MASCC serves an international community, these findings highlight the need to proactively understand the concept of consumer engagement and preferred terminologies that reflect diverse contexts.

This study was designed as a first multinational consultation with consumers to garner perceptions on terminology and areas of consumer interest to inform the establishment of an engagement strategy. For MASCC seeking initial perspectives, the communication provides an additional opportunity to raise awareness of supportive care and mutual benefits of consumers as partners to support direction and implementation of the developed strategy.

**Methods**

*Study design and setting*

This multinational, cross-sectional survey study was conducted between October 2021 and December 2021. Approval was obtained from the Survey and Behavioral Research Ethics Committee of the Chinese University of Hong Kong (Hong Kong; Reference number: SBRE-21-0018).

*Eligibility and recruitment*

Study participants were recruited using a combination of purposive, convenience and snowball sampling. To be eligible for this survey, participants identified as: (1) older than 18 years; (2) able to understand written English, and (3) a “person affected by cancer”. This term “person affected by cancer” was adopted to include, but not limited to, patients or survivors of cancer, family members, carers, users of cancer-related services, members of a cancer-related advocacy group (non-governmental organizations, cancer societies, cancer forum etc.).

*Survey instrument*

The development of the survey was led by the members of the MASCC Consumer Engagement Committee, which included consumer engagement experts (ET, JMB), oncologists (BK, ML), and supportive care researchers / scientists (AB, AC, HRW, YTC) representing North America, Europe and Asia-Pacific practice settings. The survey content was informed by existing literature on engaging with patients or/and the public [17-19], particularly in cancer-related programs, services, support activities and research [2-5, 16, 20, 21]. The survey items were developed iteratively, with input from committee members and cancer consumers. To verify accessibility for a consumer audience, the survey was reviewed by members of the Victorian Comprehensive Cancer Centre (VCCC) Alliance Consumer Network. The survey was self-administered in English and took approximately 5 minutes to complete.

The overarching aim of the survey was to seek the opinions of consumers and develop preliminary directions on strategies and mechanisms to engage people affected by cancer in MASCC. The survey comprised two sections (total 15 items). The first section collected the respondents’ demographic information and the second section focused on respondents’ (i) preferred terminology to represent consumers and community, (ii) prior experience in engaging with MASCC and other cancer-related societies/ organizations, (iii) preferred areas of contribution as a consumer, and (iv) personal barriers to involvement. The survey consisted of both closed- and open-ended responses.

*Data collection*

Participants were invited to take part in the study via a survey link shared by MASCC and other cancer organizations, as well as other forms of online communication and social media platforms (LinkedIn, Twitter, and Facebook). Information about the nature and purpose of the study was provided at the beginning of the survey and participants were informed that their completion of the survey implied their consent to participate. The survey was administered online, via SurveyMonkey. At the end of the survey, all participants were encouraged to disseminate the survey to potential respondents who fulfilled the inclusion criteria. They were also assured that their responses would be anonymous and only aggregated data would be reported.

*Data analysis*

Participant responses were retrieved electronically. The IP addresses were reviewed to confirm that no participants submitted multiple entries. Due to the scoping and exploratory nature of the study, only descriptive analysis was conducted on the quantitative data. Descriptive statistics were used to summarize responses to each question for the overall cohort, as well as stratification by age group and geographical regions. The frequency of each respondents’ top tanked preference was summarized graphically, and then aggregated by age group and geographic regions.

The open-ended responses were first reviewed by all of the investigators. The first cycle of coding involved the creation of themes and assignment of data segments to the proposed themes by one investigator (YTC). After that, two investigators (HRW and JMB) validated and cross-checked the coding independently. The final coding and themes were discussed by three investigators (YTC, HRW, and JMB) in the research team and any discrepancies were resolved by consensus.

**Results**

**Sample demographic characteristics**

A total of 343 completed responses were received. Sample characteristics are presented in Table 1. The majority were female (n=268, 78.2%) and younger than 60 years of age (n=213, 62.1%). The respondents resided in the Western Europe (n=203, 59.9%), Asia-Pacific region (n=73, 21.5%), North America (n=39, 11.5%), Eastern Europe (n=16, 4.7%), Latin America/Caribbean region (n=5, 1.5%), and Africa (n=3, 0.9%), representing 29 countries or administrative regions in total. The vast majority of the respondents were from high income countries (n=329, 95.9%).

**Terminology preferences**

Respondents were asked to select preferences for seven terms commonly associated with consumer engagement and resulted in the following order of most to least preferred: ‘patient’, ‘people affected by cancer’, ‘advocate’, ‘community’, ‘carer’, ‘public’, ‘consumer’, ‘end user’ (Figure 1a). Each term was represented across all age group categories with male respondents opting for ‘patient’ as a preferred term (Figure S1). Although ‘patient’ is the majority first preference across geographical regions, this only accounts for 49% - 67% of responses across the regions and indicates partiality towards differing terminology (Figure 1b). Taking into account all of the rankings, the terms ‘patient’ and ‘people affected by cancer’ had the highest scores (Table S1). Categorization of other suggested terms (total 92 open-ended responses) revealed phrases to be neutral or having a non-patient connotation (n=36), made reference to users of cancer services (n=6) or individuals identifying as ‘living with cancer’ (n=12), ‘survivor’ (n=19), or healthcare professionals (n=10) (Table 3). The range of responses demonstrates a necessity to vary terminology appropriate to the group aiming to engage, in addition to clarifying preference choice for specific initiative needs.

**Perspectives on current engagement with MASCC or other cancer societies**

Responses were segregated on whether individuals had previously engaged. Only 22.2% (n=76) had prior engagement with MASCC. Involvement activities included conference attendances (n=55), provision of a personal narrative (n=14) or review of patient information (n=12), where multiple options were selected. Other levels of participation included special interest groups, study groups and research publications.

Of those with previous engagement with MASCC, 90.8% found it a positive or very positive experience (Table 2). The types of benefits received (total 61 open-ended responses) were education and information (n=25), networking and collaboration (n=25), community and sense of belonging (n=5) and improving practice and guidelines (n=6) (Table 3). Across all categories was the narrative that some respondents identified the international nature of MASCC as a benefit (n=7) (Table 3).

In response to suggested areas for improved engagement and, based on effective practice observed from previous interactions with medical and cancer societies, the following key themes were identified (total 176 open-ended responses): participation at every stage of decision making (n=66), being involved with educational initiatives (n=25), interaction and open communication (n=56), information sharing and strategies to improve practice (n=18) (Table 3). Suggestions for further enhancing engagement included: promoting awareness of MASCC, raising profile of consumers within societies, and having a more visible multinational presence (n=9).

**Considerations for future engagement**

A total of 64.4% (n=221) of respondents indicated interest in receiving the results of this study. Across all geographical regions, consumers indicated a preference to contribute or be consulted in consumer research (n=122), policy (n=73) or consumer engagement activities (n=131) (Figure 2). Additional suggestions for involvement included guidelines development, improving practice, research, and training and educational activities. In relation to attendance at specific events, 47.9% (n=115) of respondents indicated interest to attend a patient day, 65.0% (n=156) to attend a conference session, and 47.9% (n=115) the entire conference. Respondents perceived potential barriers for attendance to include cost, time, workload, health, internet access and COVID-19 travel restrictions.

**Discussion**

The Multinational Association for Supportive Care in Cancer (MASCC) is committed to ensuring the lived experiences of people affected by cancer are embedded within its organizational strategy. Here, we report the results of a multinational consumer survey which highlighted the profound diversity in consumer perspectives and preferences. Through ongoing co-design, these findings will inform the development and implementation of a MASCC Model of Consumer Engagement to promote sustainable consumer partnerships and to deliver bi-directional benefits.

Consumer engagement is a challenging concept to define as it does not incorporate a single concept or type of activity [3]. Even within a particular context, consumer engagement operates along a continuum, with a constellation of opportunities for people with expertise through lived experiences to contribute or benefit through engagement. This has undoubtedly led to confusion, miscommunication and a misunderstanding of what defines consumer engagement and at what level it can be incorporated into health practices, research, policy and professional societies. At a more granular level, there is considerable variation in the terminology used to describe consumers; a strong theme identified in this survey. Of interest, the term “patient” was most commonly ranked as the preferred term by respondents; a term that has received some criticism for defining a person by their illness [15, 16]. However, this finding is consistent with a recent scoping review which identified, across 47 articles, that “patient” was the preferred term for healthcare recipients (with cancer) [16]. Combined, these data suggest that despite calls for more “politically correct” terminology and a move towards “person”-centered care, the term patient has persisted.

Preference for the term patient may reflect familiarity with the term for regular users of healthcare services, or unfamiliarity with emerging terms like “consumer”. However, it may also reflect a geographic bias in our data, with preferences reported to vary cross-culturally. For example, preferences for the term “client” over “patient” are higher in the US than other countries [16]. While our respondents were from 29 countries, almost 60% resided in Western Europe where the term “patient” is widely used [21].

When looking at only the top 3 terms (“patient”, “advocate”, “people affected by cancer”), the term “patient” accounted for ~50% of preferred responses in each geographic region. While this demonstrates consistency *across* geographic regions, it highlights variability *within* these regions. This may be driven by the range of people who may self-identify as “people affected by cancer”, each of whom resonate with a different term based on their consumer/advocacy-related activities. Interestingly, healthcare professionals that completed this survey may self-identify as a “person affected by cancer” based on either their profession (i.e. the provision of cancer care) or due to a cancer diagnosis. While our results did not enable such detailed insight into the specific characteristics of respondents, these findings do highlight a potentially overlooked consumer category - healthcare providers who are also consumers. This category draws similarities with “patient-researchers”, an increasingly recognized subset of biomedical researchers [20]. Like “patient-researchers”, “patient-healthcare providers” have combined knowledge of living with cancer, as well as the intricacies of a healthcare system. Regardless, all subcategories of consumers require careful consideration and the need for terminology to be a point of critical discussion and ongoing consultation in establishing a MASCC Model of Consumer Engagement. In reality, it is likely that different terms will be used in distinct circumstances depending on the activity and the anticipated role the consumer will play. In these circumstances it is critical that the term be clearly defined and justified.

In establishing the Model of Consumer Engagement, key principles will ensure the benefits of engagement are not one directional. Results indicating preferred methods of engagement underscore the importance of societies interacting with consumers in more substantive ways than simply the provision of patient-resources, and toward integration of consumer perspectives into the inner fabric of the organization. Of interest, this survey was also completed by people who had ***not*** previously engaged with MASCC, suggesting the existence of a consumer population that would like to engage with Societies like MASCC but has not yet done so. This may have been impacted by the range of barriers consistently identified to limit engagement, including cost, complexities of travel requirements and availability [22-24]. Appropriate recognition, particularly in a post-pandemic world, of these barriers is critical to promote strong consumer representation at annual meetings. We anticipate that via authentic co-design and implementation of a structured Model of Consumer Engagement, consumer involvement and attendance will be promoted, and indeed celebrated, enabling identified benefits of consumer engagement to be amplified and diversified. By engaging with consumers at this early stage, the insight gleaned from this survey study will be applied to inform the future consumer engagement framework of MASCC.

While the results of this survey will form a critical foundation to now develop a Model of Consumer Engagement, they must be considered in light of some limitations. Most notably, the survey was conducted in English and disseminated electronically over the internet. Hence the resultant population is dominated by English-speaking countries in the regions of Western Europe, Asia-Pacific and North America. This may limit the generalizability of results. Although we did not collect information on the respondents’ education attainment and other socioeconomic variables, it is likely that the underrepresentation of respondents from certain geographical regions, especially low- and middle-income countries, might be due to language barriers, access to the survey for completion, or relatively poorer understanding of consumer engagement and supportive care concepts. Even though the survey was disseminated on various social media platforms and through cancer organizations /networks, there might be potential sampling bias as the respondents were more likely to be cognizant of MASCC and the topic on consumer engagement. However, we also identified the lack of awareness of MASCC as one of the recurring subthemes – this finding suggested that our survey did reach individuals who had no prior connection or knowledge of MASCC to a certain extent. Future studies may investigate specific barriers in low and low-middle income settings. This emphasizes the need for Societies, Associations, and advocacy groups to identify culturally-diverse local “champions” to develop strategies that ensure equitable opportunities for consumer engagement.

In conclusion, this scoping study is the first to provide preliminary insights about consumer engagement in cancer supportive care at a multinational level. The findings are critical to inform strategies and shape future directions in engaging consumers in the delivery of cancer care. Importantly, the results define the principles that will form the basis of a MASCC Model of Consumer Engagement. These principles broadly include promoting diversity in perspectives, ensuring accessible and equitable consumer engagement, and fostering sustainable partnerships with consumers (Figure 3). In establishing this Model, we will identify successful strategies in existing frameworks in the health sector [11, 25-27], adapting to reflect the unique attributes of MASCC.

**Declarations**
*Funding:* No funding was received for conducting this study.
*Conflicts of interest/Competing interests:* All authors do not have any conflict of interest to declare that are relevant to the content of this article.
*Availability of data and material:* The data that support the findings of this study are available from the corresponding author upon reasonable request.
*Code availability:* Not applicable
*Authors' contributions:* All authors contributed to the study conception, study design, and acquisition of data. Joanne M Britto, Hannah R Wardill, and Yin Ting Cheung performed data analysis and prepared the first draft of the manuscript. All authors contributed to interpretation of the data and commented on previous versions of the manuscript. All authors read and approved the final manuscript.
*Ethics approval:* Approval was obtained from the Survey and Behavioral Research Ethics Committee of The Chinese University of Hong Kong (Hong Kong; Reference number: SBRE-21-0018).
*Consent to participate:* Completion and submission of the survey implied informed consent to participate.
*Consent for publication:* Not applicable

**Tables and Figures**

**List of tables**

Table 1: Respondent demographic

Table 2: MASCC engagement experience and benefits Table 3: Major themes and quotes

**List of figures**

Figure 1: Terminology preferences

Figure 2: Potential engagement methods

Figure 3: Principles of consumer engagement

**Supplementary figures and tables**

Supplementary Figure 1: Terminology preferences by age and gender

Supplementary Table 1: Terminology preferences ranking and scores

**References**

1. Dalton J, Chambers D, Harden M, et al. (2016) Service user engagement in health service reconfiguration: a rapid evidence synthesis. J Health Serv Res Policy 21:195-205.

2. Hall AE, Bryant J, Sanson-Fisher RW, et al. (2018) Consumer input into health care: Time for a new active and comprehensive model of consumer involvement. Health Expect 21:707-713.

3. Sarrami-Foroushani P, Travaglia J, Debono D, and Braithwaite J (2014) Key concepts in consumer and community engagement: a scoping meta-review. BMC Health Services Research 14:250.

4. Pii KH, Schou LH, Piil K, and Jarden M (2019) Current trends in patient and public involvement in cancer research: A systematic review. Health Expect 22:3-20.

5. Brett J, Staniszewska S, Mockford C, et al. (2014) Mapping the impact of patient and public involvement on health and social care research: a systematic review. Health Expect 17:637-50.

6. Boote J, Wong R, and Booth A (2015) 'Talking the talk or walking the walk?' A bibliometric review of the literature on public involvement in health research published between 1995 and 2009. Health Expect 18:44-57.

7. Hall LK, Kunz BF, Davis EV, Dawson RI, and Powers RS (2015) The cancer experience map: an approach to including the patient voice in supportive care solutions. J Med Internet Res 17:e132.

8. Tenniglo LJA, Loeffen EAH, Kremer LCM, et al. (2017) Patients' and parents' views regarding supportive care in childhood cancer. Support Care Cancer 25:3151-3160.

9. McGrath P (2013) "Receptivity": an important factor affecting supportive care provision. J Psychosoc Oncol 31:30-50.

10. National Health Service Commissioning Board. NHS England Patient and Public Voice Partners Policy. URL: <https://www.england.nhs.uk/publication/patient-and-public-voice-partners-policy/>. Accessed on March 22, 2022.

11. Patient Centered Outcomes Research Institute. The Value of Engagement. URL: <https://www.pcori.org/engagement/value-engagement>. Accessed on March 22, 2022.

12. The National Health and Medical Research Council (NHMRC). Consumer and Community Engagement. URL: <https://www.nhmrc.gov.au/about-us/consumer-and-community-engagement>. Accessed on March 22, 2022.

13. Domecq JP, Prutsky G, Elraiyah T, et al. (2014) Patient engagement in research: a systematic review. BMC Health Services Research 14:89.

14. Todd AL and Nutbeam D (2018) Involving consumers in health research: what do consumers say? Public Health Res Pract 28:2821813.

15. Torrey EF (2010) Patients, Clients, Consumers, Survivors et al: What's in a Name? Schizophrenia Bulletin 37:466-468.

16. Costa DSJ, Mercieca-Bebber R, Tesson S, Seidler Z, and Lopez A-L (2019) Patient, client, consumer, survivor or other alternatives? A scoping review of preferred terms for labelling individuals who access healthcare across settings. BMJ Open 9:e025166.

17. Natafgi N, Ladeji O, Blackwell S, et al. (2022) Similar values, different expectations: How do patients and providers view 'health' and perceive the healthcare experience? Health Expect 25:1517-1528.

18. Iliffe S and Manthorpe J (2021) Medical consumerism in the UK, from 'citizen's challenge' to the 'managed consumer'-A symbol without meaning? Health Expect 24:182-187.

19. Boivin A, Dumez V, Castonguay G, and Berkesse A (2022) The Ecology of Engagement: Fostering cooperative efforts in health with patients and communities. Health Expect.

20. Riggare S (2020) Patient researchers — the missing link? Nat Med 26:1507-1507.

21. Holmes L, Cresswell K, Williams S, et al. (2019) Innovating public engagement and patient involvement through strategic collaboration and practice. Res Involv Engagem 5:30.

22. Saunders C and Girgis A (2010) Status, challenges and facilitators of consumer involvement in Australian health and medical research. Health Res Policy Syst 8:34-34.

23. Collins K, Boote J, Ardron D, et al. (2015) Making patient and public involvement in cancer and palliative research a reality: academic support is vital for success. BMJ Support Palliat Care 5:203-6.

24. Milley K, Chima S, McIntosh JG, Ackland E, and Emery JD (2021) Long-term consumer involvement in cancer research: Working towards partnership. Health Expect 24:1263-1269.

25. Victorian Comprehensive Cancer Centre (VCCC) Alliance. Consumer Engagement. https://vcccalliance.org.au/our-work/consumer-engagement/. Accessed on March 22, 2022.

26. Clauser SB, Gayer C, Murphy E, Majhail NS, and Baker KS (2015) Patient Centeredness and Engagement in Quality-of-Care Oncology Research. J Oncol Pract 11:176-9.

27. Miller CL, Mott K, Cousins M, et al. (2017) Integrating consumer engagement in health and medical research - an Australian framework. Health Res Policy Syst 15:9.

| **Table 1 Demographics of respondents N=343** | |
| --- | --- |
|  | n (%) |
| **Age (years)** |  |
| 19 – 29 | 13 (3.8) |
| 30 – 39 | 33 (9.7) |
| 40 – 49 | 63 (18.5) |
| 50 – 59 | 104 (30.5) |
| Above 60 | 128 (37.5) |
| Preferred not to disclose | 2 (0.6) |
| **Gender** |  |
| Male | 71 (20.7) |
| Female | 268 (78.1) |
| Preferred not to disclose | 4 (1.2) |
| **Regions of the world** |  |
| Africa | 3 (0.9) |
| Asia-Pacific | 73 (21.3) |
| Eastern Europe | 16 (4.7) |
| Latin America and Caribbean | 5 (1.6) |
| Western Europe | 203 (59.2) |
| North America | 39 (11.4) |
| Preferred not to disclose | 4 (1.2) |
| **Income level (based on World Bank)** |  |
| Low-middle income countries | 14 (4.1) |
| High income countries | 329 (95.9) |

| **Table 2 Previous experience and perceived benefits** | | | |
| --- | --- | --- | --- |
|  |  | ***n*** | **%** |
| **Respondents who have engaged with MASCC (n = 76)** | | | |
| **Perceived experience** | Very positive | 34 | 44.7% |
|  | Positive | 35 | 46.1% |
|  | Neutral | 7 | 9.2% |
| **Perceived benefits** | Yes | 63 | 82.9% |
|  | No | 12 | 15.8% |
|  | No response | 1 | 1.3% |
| **Respondents who have *not* engaged with MASCC (n = 245)** | | | |
| **Perceived experience** | Very positive | 7 | 2.9% |
|  | Positive | 18 | 7.4% |
|  | Neutral | 102 | 41.6% |
|  | No response | 118 | 48.2% |
| **Perceived benefits** | Yes | 20 | 8.2% |
|  | No | 78 | 31.8% |
|  | No response | 147 | 60.0% |

| **Table 3: Themes and representative quotes from the open-ended responses** | | |
| --- | --- | --- |
| **Items** | **Themes** | **Quotes** |
| **Preferred terminologies** | A neutral, non-patient oriented connotation | · *“Individuals with cancer”*  · *“Person with cancer”* |
|  | Users of services related to cancer | · *“Service user”*  · *“Cancer service user”* |
|  | Living with cancer | · *“Survivor”*  · *“Living with and beyond cancer”*  · *“Thriving with cancer”* |
|  | Healthcare providers | · *“Healthcare professional”*  · *“Clinician”*  · *“Nurse”* |
| **Benefits from MASCC** | Education and information | · *“Improved patient education materials content”*  · *“Listening to new information”*  · *“To access information about supportive care”* |
|  | Networking and collaboration | · *“Collaborations, sharing of practice”*  · *“Meeting others in a common interest group”*  · *“Meeting people from all over the world with the same goals, optimizing care for the cancer patient”* |
|  | Community and sense of belonging | · *“Felt part of something”*  · *“Giving back from my own experience”*  · *“Meeting people who are going through the same thing”* |
|  | Guidelines and improving practice | · *“Generation of high quality guidelines”*  · *“Informative patient centered care”*  · *“Learning around supporting patients with bone metastasis”*  · *“Started my supportive oral care program for cancer patients”* |
|  | International perspectives | · *“International conference therefore interesting to listen to what others are doing overseas.”*  · *“Being able to get the perspective of cancer care through a more global lens”*  · *“It opened my eyes to what is happening around the world”*  · *“Joining the people affected by cancer and their caregivers throughout the world - together - about supportive care and link in with similar groups”* |
| **Improving the consumer experience** | Engagement and participation at every stage of decision making | ·  *“Early involvement rather than just looking for confirmation or small tweaks to something already close to finalized NOT expecting the consumers to spend most of their time educating the committee on how to involve consumers as opposed to providing substantive contributions”*  · *“Involving community representatives, patients, and caregivers with materials development or content creation at the planning and evaluation stages*  · *“Sharing of ideas, joint research, peer support, improved patient outcomes, inclusiveness”*  · *“Equal and respectful collaboration. Recognizing the people with a cancer diagnosis have real lives and bring valid ideas, thoughts and solutions to the challenges of cancer”* |
|  | Being involved in educational initiatives | · *“Education and seminar of cancer care in community practice”*  · *“The information for people to acknowledge their health issues and how to manage their symptoms”*  · *“A focus on short, easy reading patient education materials available in multiple languages”* |
|  | Interaction and open communication | · *Social media to communicate”*  · *“Proximity and allow to engage with other people in your same situation”*  · *“Open lines of communication between patients and medical professionals”*  · *“Knowing the families on a personal level, knowing the interest of the child and being able to make consecration around this interest”* |
|  | Sharing information and strategies to improve practice and care | · *“Shared best practice and justifying practice either for change or to maintain current services”*  · *“Attention to complementary therapies”* |
|  | Promoting awareness | ·  *“Raising profile to wider audience”*  · *“More active / visible / scientific presence in global scientific congress dealing with the treatment of cancer”*  · *“Collaborate with major cancer organizations on health literacy issues”* |

**
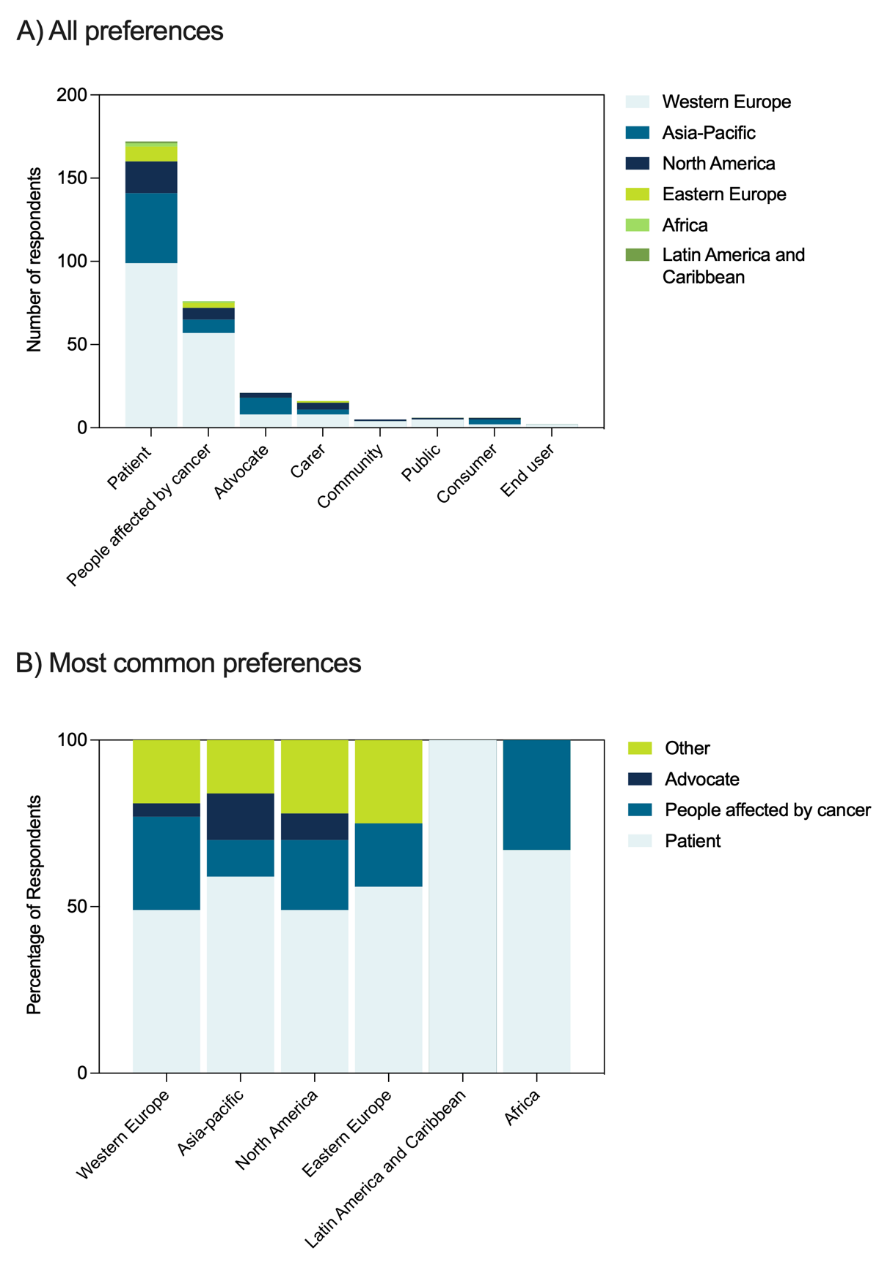
**

**Figure 1: Terminology preferences. (A)** Overall top-ranked preferred terminologies in absolute numbers (*n*), stratified by geographical regions. **(B)** Preferences for the most common terminologies in proportions (%), stratified by geographical regions. Missing entries were excluded from the analyses.

**
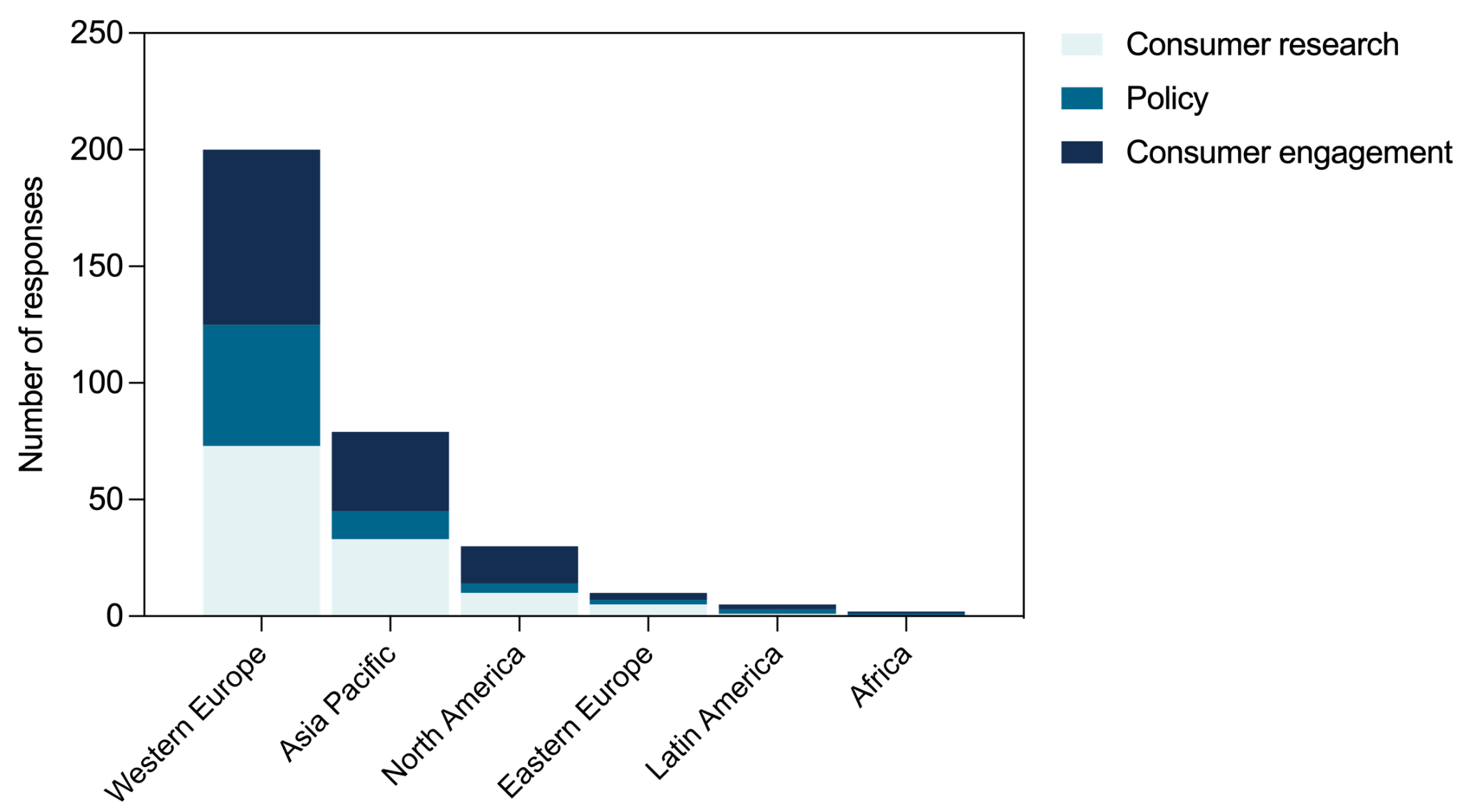
**

**Figure 2: Potential engagement methods.** The number of respondents indicating preferences for each engagement method, stratified by geographical regions


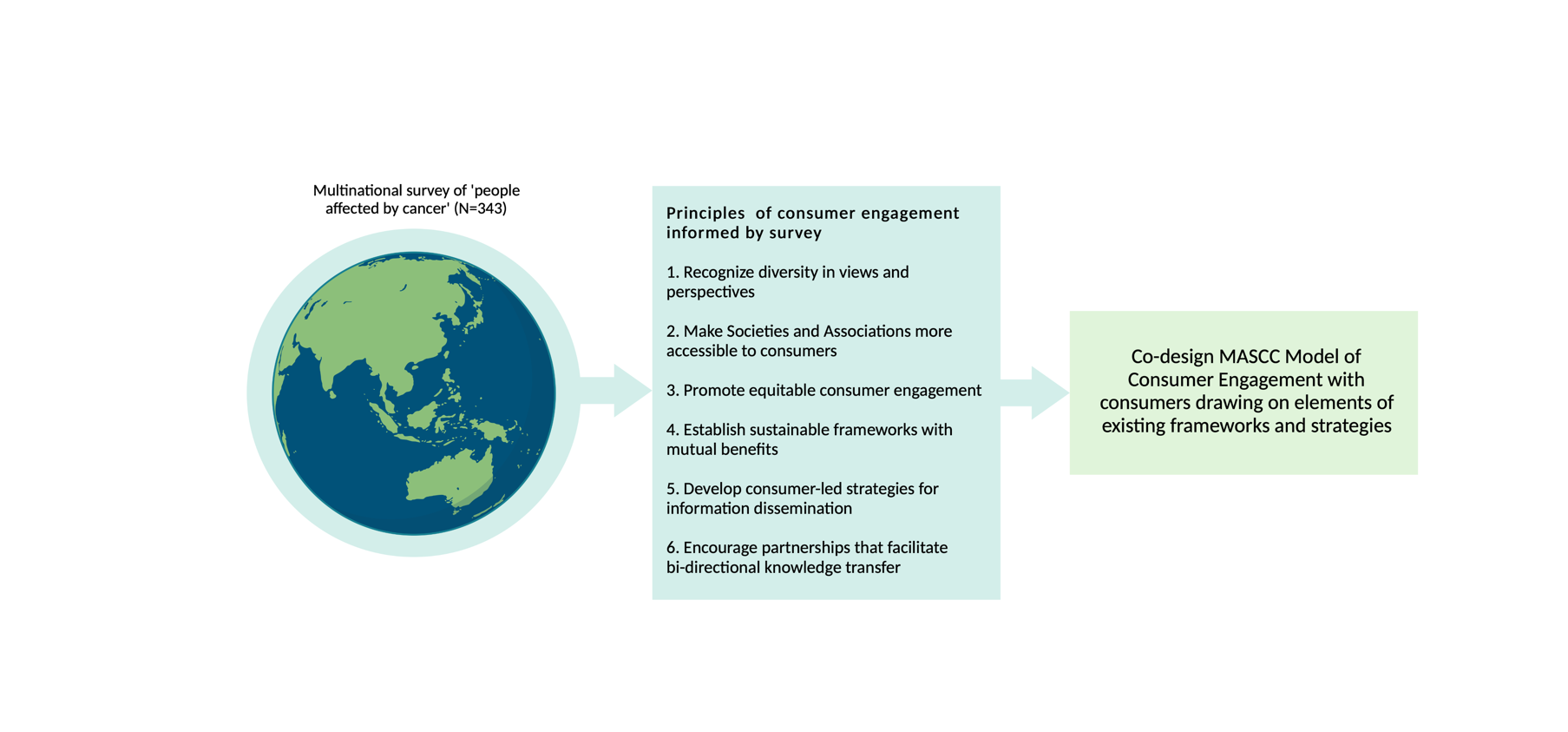


**Figure 3: Principles of consumer engagement.** Based on the findings of this survey study and collective evidence from the literature, six principles are proposed to guide the development of the MASCC Model of Consumer Engagement.

**Figure S1: Terminology preferences categorized by (A) age and (B) gender.** **(A)** Overall preferred terminologies in absolute numbers (*n*), stratified by age. **(B)** Overall preferred terminologies in absolute numbers (*n*), stratified by gender.


| **Table S1: Terminology preferences ranking and scores** | | | | | | | | | | |
| --- | --- | --- | --- | --- | --- | --- | --- | --- | --- | --- |
| **Preference** | **1** | **2** | **3** | **4** | **5** | **6** | **7** | **8** | **TOTAL** | **SCORE** |
| **Patient** | 58.64%  173 | 16.95%  50 | 5.42%  16 | 5.76%  17 | 2.37%  7 | 3.39%  10 | 3.05%  9 | 4.41%  13 | 295 | 6.79 |
| **Consumer** | 2.31%  6 | 14.23%  37 | 10.00%  26 | 11.54%  30 | 5.77%  15 | 12.31%  32 | 23.46%  61 | 20.38%  53 | 260 | 3.63 |
| **End user** | 0.78%  2 | 2.35%  6 | 9.02%  23 | 7.45%  19 | 10.20%  26 | 9.02%  23 | 23.53%  60 | 37.65%  96 | 255 | 2.67 |
| **Community** | 1.92%  5 | 9.23%  24 | 19.62%  51 | 21.15%  55 | 22.31%  58 | 17.31%  45 | 6.15%  16 | 2.31%  6 | 260 | 4.59 |
| **People affected by cancer** | 26.55%  77 | 35.17%  102 | 14.48%  42 | 6.90%  20 | 7.93%  23 | 3.10%  9 | 2.76%  8 | 3.10%  9 | 290 | 6.30 |
| **Public** | 2.32%  6 | 5.41%  14 | 9.27%  24 | 14.67%  38 | 20.08%  52 | 29.34%  76 | 11.58%  30 | 7.34%  19 | 259 | 3.84 |
| **Advocate** | 7.78%  21 | 14.44%  39 | 20.00%  54 | 14.07%  38 | 15.93%  43 | 10.00%  27 | 12.96%  35 | 4.81%  13 | 270 | 4.78 |
| **Carer** | 6.04%  16 | 6.79%  18 | 15.09%  40 | 15.85%  42 | 11.32%  30 | 13.21%  35 | 13.21%  35 | 18.49%  49 | 265 | 3.95 |
